# Phylogeny and divergence of the pinnipeds (Carnivora: Mammalia) assessed using a multigene dataset

**DOI:** 10.1186/1471-2148-7-216

**Published:** 2007-11-09

**Authors:** Jeff W Higdon, Olaf RP Bininda-Emonds, Robin MD Beck, Steven H Ferguson

**Affiliations:** 1 Department of Geography, University of Manitoba, 501 University Crescent, Winnipeg, Manitoba, R3T 2N6, Canada; 2 Institut für Spezielle Zoologie und Evolutionsbiologie mit Phyletischem Museum, Friedrich-Schiller-Universität Jena, Erbertstrasse 1, 07743 Jena, Germany; 3 School of Biological, Earth and Environmental Sciences, University of New South Wales, Sydney NSW 2052, Australia; 4 Fisheries and Oceans Canada, 501 University Crescent, Winnipeg, Manitoba, R3T 2N6, Canada

## Abstract

**Background:**

Phylogenetic comparative methods are often improved by complete phylogenies with meaningful branch lengths (e.g., divergence dates). This study presents a dated molecular supertree for all 34 world pinniped species derived from a weighted matrix representation with parsimony (MRP) supertree analysis of 50 gene trees, each determined under a maximum likelihood (ML) framework. Divergence times were determined by mapping the same sequence data (plus two additional genes) on to the supertree topology and calibrating the ML branch lengths against a range of fossil calibrations. We assessed the sensitivity of our supertree topology in two ways: 1) a second supertree with all mtDNA genes combined into a single source tree, and 2) likelihood-based supermatrix analyses. Divergence dates were also calculated using a Bayesian relaxed molecular clock with rate autocorrelation to test the sensitivity of our supertree results further.

**Results:**

The resulting phylogenies all agreed broadly with recent molecular studies, in particular supporting the monophyly of Phocidae, Otariidae, and the two phocid subfamilies, as well as an Odobenidae + Otariidae sister relationship; areas of disagreement were limited to four more poorly supported regions. Neither the supertree nor supermatrix analyses supported the monophyly of the two traditional otariid subfamilies, supporting suggestions for the need for taxonomic revision in this group. Phocid relationships were similar to other recent studies and deeper branches were generally well-resolved. *Halichoerus grypus *was nested within a paraphyletic *Pusa*, although relationships within Phocina tend to be poorly supported. Divergence date estimates for the supertree were in good agreement with other studies and the available fossil record; however, the Bayesian relaxed molecular clock divergence date estimates were significantly older.

**Conclusion:**

Our results join other recent studies and highlight the need for a re-evaluation of pinniped taxonomy, especially as regards the subfamilial classification of otariids and the generic nomenclature of Phocina. Even with the recent publication of new sequence data, the available genetic sequence information for several species, particularly those in *Arctocephalus*, remains very limited, especially for nuclear markers. However, resolution of parts of the tree will probably remain difficult, even with additional data, due to apparent rapid radiations. Our study addresses the lack of a recent pinniped phylogeny that includes all species and robust divergence dates for all nodes, and will therefore prove indispensable to comparative and macroevolutionary studies of this group of carnivores.

## Background

The pinnipeds are a monophyletic group of aquatic carnivores most closely related to either mustelids or ursids. The three monophyletic families – Phocidae (earless or true seals), Otariidae (sea lions and fur seals), and Odobenidae (one extant species of walrus) last shared a common ancestor within arctoid carnivores > 25 million years ago (mya) [1,2]. Some morphological studies [3,4] and virtually all molecular studies [e.g., [5-15]] support a link between otariids and odobenids (Otarioidea). However, several morphologists prefer a phocid-odobenid clade (e.g. [2,16-18]).

There are 34 extant species of pinniped, including *Monachus tropicalis *(which is widely believed to have gone extinct recently) and treating *Zalophus *as being monotypic (*Z. californianus*) (Table 1). The family Phocidae contains 19 species in two subfamilies: Monachinae or "southern" hemisphere seals (nine species comprising Antarctic, elephant, and monk seals) and Phocinae or "northern" hemisphere seals (10 species that inhabit the Arctic and sub-Arctic) [19]. Some authors have questioned the monophyly of Monachinae [20-22], although recent studies have shown this subfamily to be monophyletic [4,11,14,15,23,24]. The monophyly of Phocinae has not been questioned since being established by King [25]; however, there remains considerable debate over inter-group relationships, especially within Phocina (reviewed by [11,26]). The family Otariidae contains 14 extant species that have been divided traditionally into the subfamilies Arctocephalinae (fur seals) and Otariinae (sea lions) (e.g. [27,28]). Early suggestions that this subfamilial classification might be incorrect (e.g. [29]) have received increasing support from recent molecular analyses [12,14,15,30-32]. Taken together with a number of reports of both interspecific and intergeneric hybrids within Otariidae (e.g. [19,33,34]), a reassessment of otariid taxonomy based on additional phylogenetic evidence is needed. Brunner [31] provides an extensive review of the history of otariid classification. Finally, Odobenidae today comprises only the single species of walrus, *Odobenus rosmarus*.

**Table 1:** Indented taxonomy listing the 34 pinniped taxa (including the extinct *Monachus tropicalis*) included in the analyses.

Pinnipedia				
	Odobenidae			Walruses
			*Odobenus rosmarus*	Walrus
	Otariidae			Sea lions and fur seals
		Callorhinae		
			*Callorhinus ursinus*	Northern Fur Seal
		Arctocephalinae/Otariinae		
			*Arctocephalus townsendi*	Guadalupe Fur Seal
			*Arctocephalus philippii*	Juan Fernandez Fur Seal
			*Arctocephalus galapagoensis*	Galapagos Fur Seal
			*Arctocephalus australis*	South American Fur Seal
			*Arctocephalus tropicalis*	Subantarctic Fur Seal
			*Arctocephalus gazella*	Antarctic Fur Seal
			*Arctocephalus forsteri*	New Zealand Fur Seal
			*Arctocephalus pusillus*	South African Fur Seal
			*Zalophus californianus*	California Sea Lion
			*Phocarctos hookeri*	Hooker's Sea Lion
			*Neophoca cinerea*	Australian Sea Lion
			*Otaria byronia*	Southern Sea Lion
			*Eumetopias jubatus*	Northern Sea Lion
	Phocidae			True seals
		Monachinae		"Southern" true seals
		Monachini		Monk seals
			*Monachus schauinslandi*	Hawaiian Monk Seal
			*Monachus tropicalis*†	Caribbean Monk Seal
			*Monachus monachus*	Mediterranean Monk Seal
		Miroungini		Elephant seals
			*Mirounga angustirostris*	Northern Elephant Seal
			*Mirounga leonina*	Southern Elephant Seal
		Lobodontini		Antarctic seals
			*Lobodon carcinophagus*	Crabeater Seal
			*Leptonychotes weddellii*	Weddell Seal
			*Hydrurga leptonyx*	Leopard Seal
			*Ommatophoca rossii*	Ross Seal
		Phocinae		Northern true seals
		Erignathini		<no common name>
			*Erignathus barbatus*	Bearded Seal
		Cystophorini		<no common name>
			*Cystophora cristata*	Hooded Seal
		Phocini		<no common name>
		Histriophocina		<no common name>
			*Histriophoca fasciata*	Ribbon Seal
			*Pagophilus groenlandicus*	Harp Seal
		Phocina		<no common name>
			*Phoca largha*	Largha Seal
			*Phoca vitulina*	Harbor Seal
			*Pusa hispida*	Ringed Seal
			*Pusa sibirica*	Baikal Seal
			*Pusa caspica*	Caspian Seal
			*Halichoerus grypus*	Grey Seal

Several recent genetic studies [11,12,14,15,24,26,32] have advanced our knowledge of relationships within Pinnipedia considerably. Unfortunately, many of these (the exceptions being [14,24,26]) did not include divergence-date estimates as required for some types of macroevolutionary studies and phylogenetic comparative analyses. In addition, none included all species. The only study to include divergence-date estimates for all extant pinnipeds was that of Bininda-Emonds et al. [23] as a part of a larger carnivore supertree. This tree has been used extensively in comparative studies of carnivores in general (e.g., [35-42]) and pinnipeds in particular (e.g., [43-47]). However, it remains that the carnivore supertree is nearly a decade old and might no longer reflect current phylogenetic opinion.

Our objective is to address the lack of a recent phylogenetic study that includes all extant pinniped species and to provide date estimates for all nodes. To accomplish this task, we used the supertree method matrix representation with parsimony (MRP, [48,49]) to derive a complete phylogeny of the group from 50 gene trees (with mtDNA markers analyzed either individually or combined as a single source tree), with corresponding maximum likelihood (ML) and Bayesian (BI) analyses of the concatenated supermatrix serving as a form of topological sensitivity analysis in a global congruence framework [50]. Divergence dates within the supertree topology were estimated using 52 genes calibrated with eight robust fossil dates using two different methods. Together, the use of a larger data set focussed exclusively on the pinnipeds should yield both a more accurate topology and divergence dates than those present in the global carnivore supertree of Bininda-Emonds et al. [23].

## Results and Discussion

### General structure of the supertree

**Figure 1:**
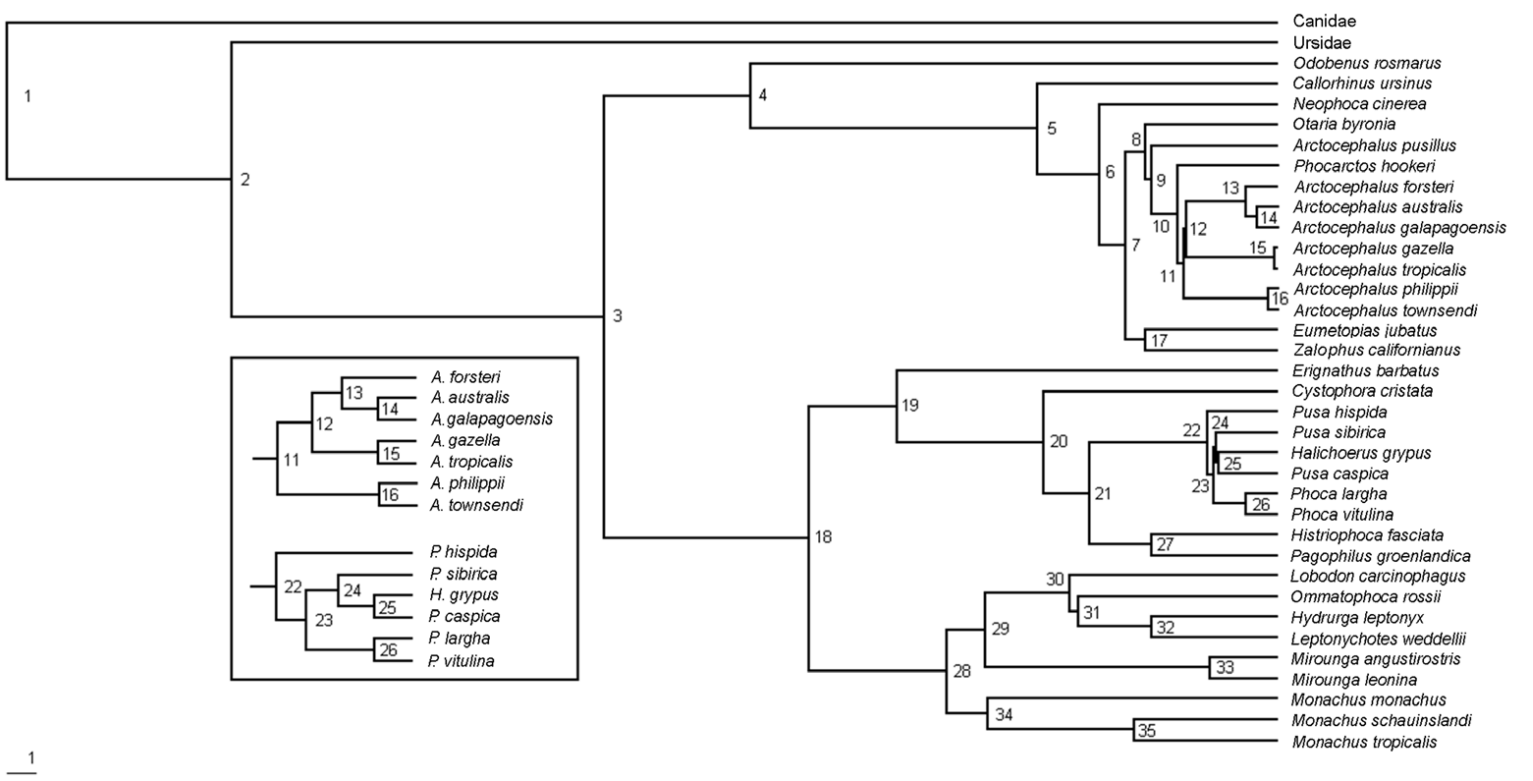
Molecular supertree of the world's extant pinnipeds (plus one recently extinct *Monachus *species) based on a weighted matrix representation with parsimony analysis of 50 maximum likelihood gene trees. Node numbers correspond to divergence dates in Table 2. Branch lengths correspond to time with the scale bar indicating one million years. Boxed subset provides additional detail on branching order for two parts of the supertree where divergences occurred over a short timeframe.

Our preferred hypothesis of pinniped evolution is that derived from the molecular supertree with all genes analyzed individually (Fig. 1; see Methods). It agrees broadly with other recent studies (e.g., [10-15,23,24,26,32]). In particular, the monophyly of each of Pinnipedia, Otarioidea, Phocidae, Otariidae, and the two phocid subfamilies was supported. Many of these nodes are among the most strongly supported in the supertree. The high level of congruence across numerous studies using different data sources and methodologies would suggest that higher-level pinniped relationships are well resolved. However, many relationships closer to the tips of the tree, particularly those within each of *Arctocephalus *and Phocina, remain contentious.

Support values within the supertree (Table 2) were generally much higher than values typically reported for the supertree-specific support measure rQS (see [51,52]), with an average rQS value (± SD) across the tree of 0.234 ± 0.214. As such, most nodes are directly supported by a majority of the 50 source trees containing all the relevant taxa. The only exception is the node comprising *Halichoerus grypus*, *Pusa caspica *and *Pusa sibirica*, which has a slightly negative rQS value (-0.040). Even so, all more inclusive nodes possess positive rQS values, indicating that the conflict has more to do with the exact placement of *Halichoerus *within *Pusa *rather than the placement of it within this genus *per se*.

**Table 2:** Divergence dates for the world's pinnipeds based on the median of up to 52 relative molecular and/or one fossil date analyzed using the relDate method.

				Confidence Interval		Number of date estimates		
				
Node	rQS	Input date	Corrected date (SE)	Lower	Upper	Total	Molecular	Fossil
1	n/a	43.35	43.4			1	0	1
2	n/a	35.7	35.7 (2.63)	30.56	40.85	14	13	1
3	0.60	23	23 (1.36)	20.33	25.67	27	26	1
4	0.12	18	18 (1.40)	15.25	20.75	16	15	1
5	0.42	8.22	8.2 (2.09)	4.12	12.32	5	4	1
6	0.36	6.11	6.1			1	1	0
7	0.36	5.15	5.2 (1.09)	3.01	7.30	16	16	0
8	0.12	4.36	4.5 (0.21)	3.95	4.77	12	12	0
9	0.20	4.36	4.3			1	1	0
10	0.20	3.21	3.4 (0.34)	2.55	3.88	2	2	0
11	0.20	2.46	3.2			1	1	0
12	0.20	3.96	3.1 (3.43)	-2.76	10.68	3	3	0
13	0.20	1.05	1.1 (0.25)	0.55	1.55	12	12	0
14	0.02	0.74	0.7			1	1	0
15	0.02	0.13	0.1			1	1	0
16	0.02	0.32	0.3			1	1	0
17	0.06	4.52	4.5 (0.37)	3.79	5.24	5	5	0
18	0.50	16	16 (0.93)	14.18	17.82	23	22	1
19	0.36	12.96	13 (0.90)	11.20	14.72	12	12	0
20	0.42	7.97	8 (0.42)	7.15	8.78	12	12	0
21	0.26	6.4	6.4 (0.40)	5.62	7.18	13	13	0
22	0.38	2.29	2.4 (0.23)	1.84	2.73	12	12	0
23	0.10	2.2	2.2 (0.62)	0.99	3.41	18	18	0
24	-0.04	2.2	2.1 (0.21)	1.79	2.61	3	3	0
25	0.00	1.99	2 (0.14)	1.71	2.27	3	3	0
26	0.12	1.07	1.1 (0.18)	0.71	1.43	12	12	0
27	0.02	4.34	4.3 (0.51)	3.35	5.33	5	5	0
28	0.22	11.33	11.3 (0.60)	10.16	12.51	15	14	1
29	0.18	9.97	10 (0.65)	8.69	11.25	21	20	1
30	0.30	7.07	7.1 (0.34)	6.41	7.73	16	16	0
31	0.06	6.81	6.8 (0.26)	6.29	7.32	17	17	0
32	0.34	4.32	4.3 (0.55)	3.24	5.39	21	21	0
33	0.32	2.28	2.3 (0.85)	0.61	3.96	21	21	0
34	0.08	9.95	9.9 (0.28)	9.40	10.49	12	12	0
35	n/a	4.9	4.9			0	0	0

Node numbers correspond to Figure 1. Dates and 95% confidence intervals are in millions of years ago, with the latter applying to the input dates only. Fossil dates correspond to those listed in Table 5 and act as constraints on the minimum divergence time for the node in question. The date for node 35 was interpolated according to a constant birth model (see Methods). Support values for each node, as measured by rQS [51, 52] are also provided.

**Figure 2:**
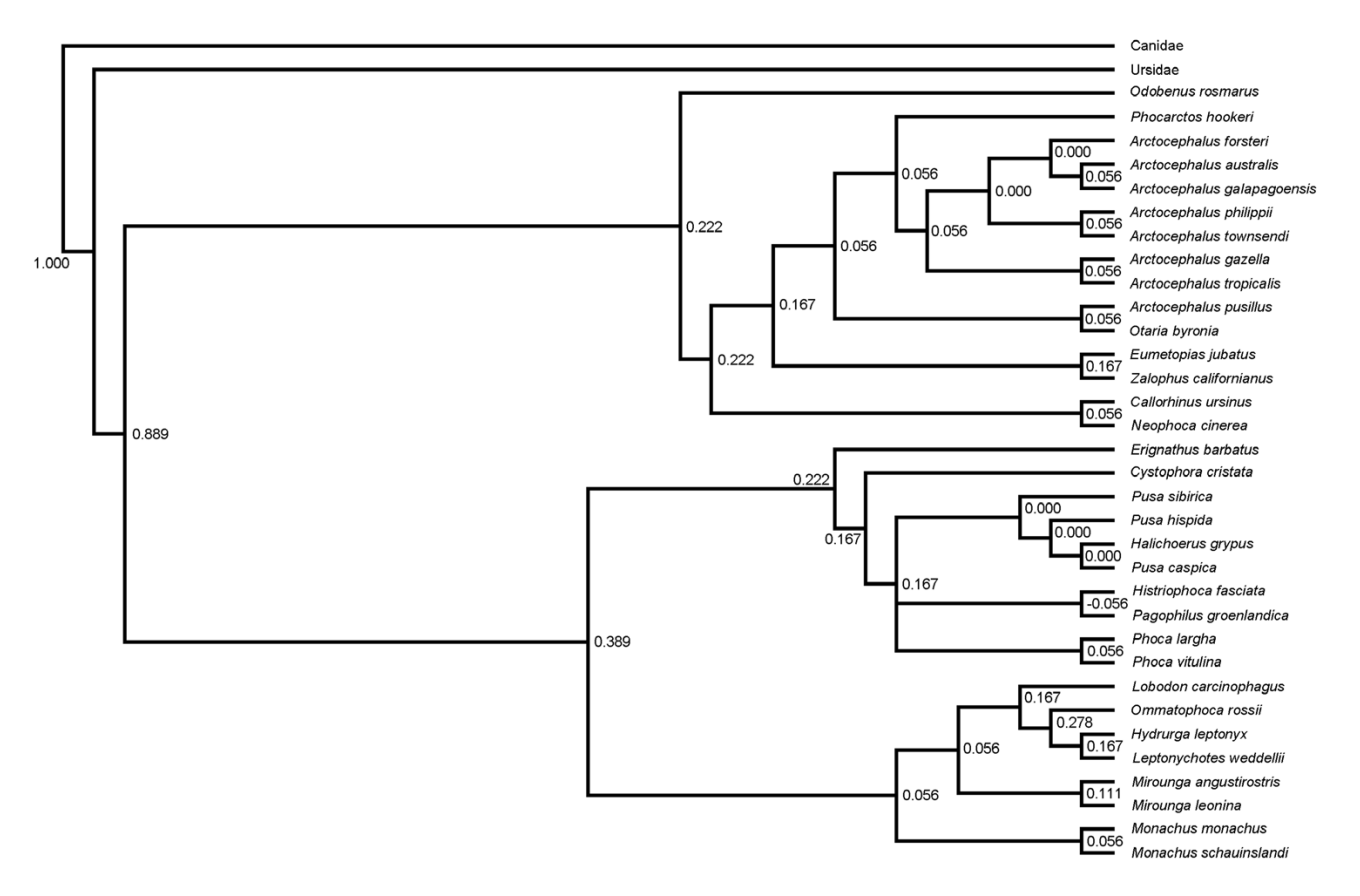
Molecular supertree of the world's extant pinnipeds (excluding the recently extinct *Monachus tropicalis*) based on a weighted matrix representation with parsimony analysis of 12 maximum likelihood gene trees, where all mtDNA genes were combined to form a single source tree. Support values for each node, as measured by rQS [51, 52] are also provided.

**Figure 3:**
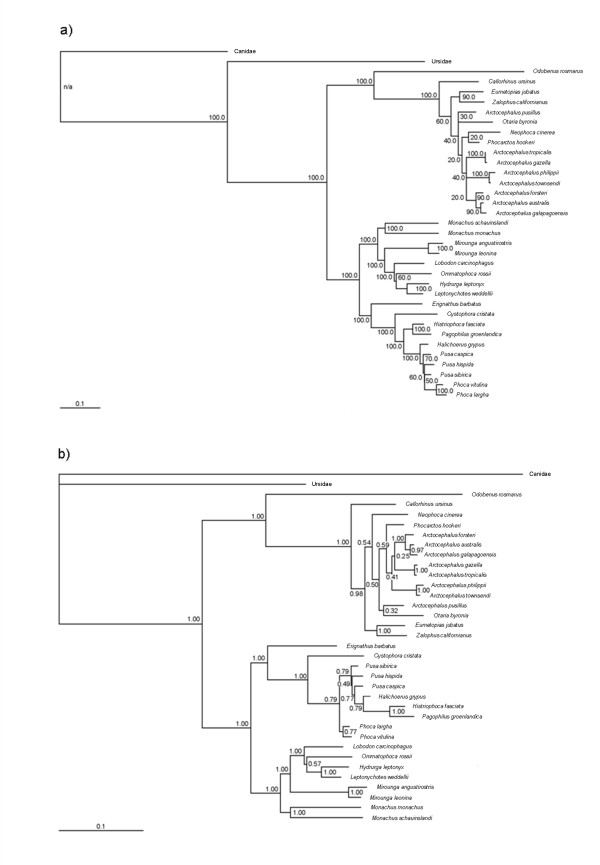
Likelihood-based analyses of the molecular supermatrix of 50 gene trees: a) ML tree with bootstrap proportions and b) BI tree with posterior probabilities. Scale bars indicate average number of substitutions per site per unit time.

Alternative analyses of the molecular data set (supertree analysis with all mtDNA forming a single source tree or ML or BI analyses of the combined supermatrix; Figures 2 and 3, respectively) yield topologies that agree broadly with that in Figure 1. The rQs support measure across the supertree (0.18 ± 0.11) again showed that most nodes are directly supported by a majority of the 12 source trees containing all the relevant taxa. In all cases, the changes occur in parts of the tree with noticeably weaker support and/or branch lengths, indicating general regions of uncertainty: 1) *Neophoca cinerea *nests deeper within otariids, either as the sister taxon to *Phocarctos hookeri *(ML) or to the clade comprising the genera *Arctocephalus*, *Otaria*, and *Phocarctos *(BI), or forms the sister taxon to *Callorhinus ursinus *(supertree); 2) the formation of a sister-group relationship between *Otaria byronia *and *Arctocephalus pusillus*, which were previously adjacent to one another (all analyses); 3) the clades (*Arctocephalus townsendii *+ *A*. *phillippi*) and (*A*. *gazella *+ *A*. *tropicalis*) trade places (all analyses); and 4) changes to the internal relationships of Phocina, either with *Halichoerus grypus *and *Pusa caspica *being pulled basally with respect to the remainder of the group, with *Halichoerus *forming the sister group to the remaining species (ML), or with *Pusa hispida *and the clade of *Histriophoca fasciata *and *Pagophilus groenlandicus *nesting deeper within the group (BI), or with *Pusa hispida *moving inside *P*. *sibirica *and with a polytomy at the base of Phocini (supertree).

In the supertree, nodes 1 and 2 (see Fig. 1) represent the divergences of the canid and ursid lineages, respectively, and nodes 3 to 35 represent the various pinniped divergences. The total sample size (molecular and fossil date estimates) underlying the divergence times for each node ranged from 0 (node 35 – the split between *Monachus schauinslandi *and *M. tropicalis*, where the date was interpolated using a constant birth model) to 27 (Table 2). Over half (19) of the pinniped nodes were dated using at least 12 separate estimates. The remaining 14 nodes were dated by five or fewer estimates. Ten of these 14 nodes relate to otariid relationships, and seven concern *Arctocephalus *species. Divergences within the *Pusa *+ *Halichoerus *clade were also dated by a comparatively small number of estimates. However, no obvious relationship existed between the variability in a date estimate (given by the coefficient of variation, CV) and the number of estimates it was derived from (R^2 ^= 0.02, *P *= 0.4849, *df *= 26).

Our inferred relDate dates for the supertree topology (see Methods) are also significantly correlated with those for comparable nodes (which are restricted largely to Phocidae) in the two major studies to estimate divergence times within pinnipeds, those of Bininda-Emonds et al. [23] (*R*^2 ^= 0.52, *P *= 0.004) and Arnason et al. [14] (*R*^2 ^= 0.958, *P *< 0.0001) (*df *= 12 in both cases using ln-transformed values). However, whereas our dates did not differ significantly from those of Bininda-Emonds et al. [23] (paired-*t *of ln-transformed values = -1.36, *P *= 0.197; *df *= 13), they were significantly more recent than those of Arnason et al. [14] (paired-*t *of ln-transformed values = -9.82, *P *< 0.0001; *df *= 13), probably reflecting their use of a only single and more distant calibration point (the caniform-feliform split at 52 mya) as well as topological differences between the trees and different methodologies used to derive the dates.

Both sets of multidivtime divergence dates (Table 3) are significantly different from the relDate divergence dates (paired-*t *of ln-transformed values = -11.39, *P *< 0.0001; *df *= 32, for relDate versus multidivtime all genes; paired-*t *of ln-transformed values = -4.53, *P *< 0.0001; *df *= 32, for relDate versus multidivtime mtDNA only). The supertree (relDate) divergence dates underestimate the multidivtime dates from all genes and mtDNA genes by 88% and 51% on average, respectively. With respect to confidence intervals (CIs), only 9 and 7 (of 33) of the relDate dates fall into the range provided by the multidivtime CIs for mtDNA or all genes, respectively. Conversely, only 3 and 4 (of 33) dates for all genes and mtDNA only, respectively, fall within the CIs of the relDate dates. However, it is important to note that the two sets of multidivtime dates themselves are also significantly different from one another (paired-*t *of ln-transformed values = 2.36, *P *= 0.02; *df *= 32). In the following sections, we compare both sets of divergence dates (i.e., the relDate and multidivtime dates) with those from the fossil record and other studies.

**Table 3:** Divergence dates calculated using Bayesian relaxed molecular clock method implemented by multidivtime [122, 123] for all genes combined and for mtDNA genes only, each fitted to the preferred supertree topology (Fig. 1).

	MultiDivTime dates (rttm = 1.95; bigtime = 4.335)							
	
	All gennes				mtDNA genes only			
		
Node	Point estimate	1 SD	95% CI (lower)	95% CI (upper)	Point estimate	1 SD	95% CI (lower)	95% CI (upper)
1								
2	35.27	3.53	29.91	42.52	36.34	3.39	30.61	42.74
3	26.67	2.64	23.15	32.44	26.73	2.36	23.28	31.86
4	21.67	2.27	18.47	26.77	21.16	2.01	18.29	25.77
5	11.91	1.98	8.18	16.05	10.72	1.84	7.45	14.66
6	9.98	1.86	6.58	13.94	9.27	1.71	6.29	12.98
7	9.16	1.76	6.00	12.89	8.72	1.64	5.87	12.25
8	7.35	1.54	4.67	10.68	6.86	1.39	4.50	9.96
9	7.07	1.50	4.46	10.32	6.58	1.36	4.29	9.59
10	5.98	1.43	3.54	9.11	5.56	1.29	3.41	8.43
11	4.87	1.21	2.86	7.58	4.58	1.08	2.81	6.99
12	4.63	1.17	2.69	7.26	4.34	1.05	2.64	6.68
13	2.02	0.63	1.07	3.51	1.91	0.54	1.09	3.21
14	0.95	0.55	0.11	2.24	0.90	0.51	0.11	2.07
15	0.50	0.40	0.03	1.51	0.02	0.02	0.00	0.07
16	0.79	0.59	0.07	2.32	0.70	0.49	0.06	1.96
17	6.57	1.50	3.98	9.83	6.10	1.37	3.81	9.14
18	22.22	2.33	18.95	27.40	21.37	2.00	18.56	26.01
19	19.89	2.21	16.57	24.84	18.63	1.85	16.15	22.98
20	14.45	1.93	11.21	18.72	12.53	1.62	9.87	16.21
21	12.68	1.85	9.51	16.75	10.93	1.56	8.33	14.44
22	6.86	1.48	4.37	10.15	4.48	1.04	2.85	6.90
23	6.47	1.41	4.10	9.62	4.05	0.95	2.56	6.26
24	6.06	1.36	3.78	9.12	3.77	0.91	2.36	5.89
25	5.46	1.29	3.33	8.35	3.31	0.83	2.03	5.26
26	2.11	0.56	1.23	3.40	1.75	0.46	1.04	2.82
27	8.34	1.66	5.45	11.93	7.40	1.41	5.01	10.53
28	18.16	2.23	14.54	23.15	16.80	1.92	13.76	21.21
29	16.54	2.19	12.82	21.45	15.05	1.87	11.97	19.30
30	13.43	2.09	9.78	18.05	11.91	1.75	8.96	15.82
31	12.92	2.07	9.30	17.51	11.41	1.72	8.52	15.29
32	8.93	1.77	5.84	12.86	7.47	1.40	5.14	10.63
33	4.64	1.49	2.47	8.24	3.45	0.97	2.07	5.82
34	16.25	2.22	12.44	21.17	14.98	1.91	11.81	19.27
35	n/a	n/a	n/a	n/a	n/a	n/a	n/a	n/a

### Origins of major pinniped groups

The split between ursids and pinnipeds is estimated to be 35.7 ± 2.63 (= mean ± SE) mya (relDate, Table 2; the multidivtime dates for this node were similar (Table 3)), although this should not be taken to imply that ursids are the closest living relatives of pinnipeds among arctoid carnivores. Early pinnipeds (pinnipedimorphs) are held to have originated in the North Pacific during the late Oligocene (34-24 mya) ([2,22,45,53], but see [14], who speculate on an origin on the southern shores of North America), which is consistent with our estimate. Thereafter, a substantial lag is apparent, with the basal pinniped split between Phocidae and Otarioidea occurring some 12 million years later at 23.0 ± 1.36 mya (Table 2) (ca. 26 mya with multidivtime, Table 3). Both values are more recent than the 28.1 mya and 33.0 mya estimates obtained by Bininda-Emonds et al. [23] and Arnason et al. [14], respectively.

Odobenidae includes a single extant species and at least 20 fossil species in 14 genera [2], with the most basal taxa known from the late early Miocene (ca. 21-16 mya). Deméré et al. [2] suggest that odobenoids first evolved in the North Pacific region sometime before 18 mya (late early Miocene), and our data indicate the upper bound to be 20.8 mya. The multidivtime dates were similar at ca. 21 mya. Both values are substantially older than the 14.2 mya estimate obtained by Bininda-Emonds et al. [23], but younger than the 26.0 mya estimate of Arnason et al. [14].

Modern fur seals and sea lions are thought to have evolved from the ancestral family Enaliarctidae ca. 11 mya [54-56], with our data showing that the diversification of the crown group occurred shortly thereafter at 8.2 ± 2.09 mya (the dates estimated using multidivtime were again older, ca. 11 mya). Arnason et al. [14] consider the late Oligocene Enaliarctinae [57] to be the oldest otarioid lineage so far described (25–27 mya; [58]). However, Deméré et al. [2] consider this group to be early pinnipedimorphs that originated before the evolution of the modern crown-group pinnipeds.

The first phocid fossils date from the middle Miocene (ca. 16-14 mya) (but see [59,60]) in the North Atlantic [61], although some authors (e.g., [2,4,62]) have speculated over a North Pacific origin. Koretsky and Sanders [59,60] recently described the "Oligocene seal" from the late Oligocene (ca. 28 mya) in South Carolina as the oldest known true seal, a fossil that predates our estimate for the basal-most split in all pinnipeds. However, because this new description was based on a very small sample (two partial femora), and because Deméré et al. [2] noted that its stratigraphic provenience may be in question, we instead used 23 mya as a conservative fossil calibration point for the split between Phocidae and Otarioidea. Obviously, acceptance of the "Oligocene seal" as the oldest known phocid (and therefore crown-group pinniped) would cause all divergence times within the pinnipeds to be older than the ones that we report.

### Otariidae

#### Phylogeny

The supertree resolved *Callorhinus ursinus *as sister to all remaining otariids (as is now generally accepted [12-14,23,32]), with neither the sea lions nor *Arctocephalus *forming clades. The five sea lion genera were generally positioned basally to the various *Arctocephalus *species. The exception was *Phocarctos *(and possibly *Otaria *in the supermatrix analyses), which nested within *Arctocephalus*. Wynen et al. [32] also reconstructed *Neophoca *as being the next otariid species to diverge (contra the supermatrix results) and found *Zalophus + Eumetopias *to form the sister clade to the remaining forms (*Arctocephalus*, *Otaria *and *Phocarctos*). These results add to the already large body of evidence, both molecular and morphological, that subfamilial descriptions in Otariidae, traditionally based on the single character of presence or absence of underfur, are incorrect [7,12,14,15,30-32,53,63]. However, resolution of most of the more inclusive otariid clades remains problematic [14,15,32], especially the relationships among the various *Arctocephalus *species, and the placements of the *A*. *australis *+ *A. forsteri *+ *A. galapagoensis *and *A*. *philippii *+ *A*. *townsendi *clades in particular. The likelihood-based supermatrix analyses reinforce the generally weak or conflicting phylogenetic signal in the data set for otariids, with both suggesting what is to our knowledge a novel, more nested position for *Neophoca *(although the inferred location differs between the analyses).

The supertree resolved *A. forsteri *as the sister to *A. australis *+ *A. galapagoensis*, with all three as sister to an *A. gazella *+ *A. tropicalis *clade, an arrangement with relatively moderate support (Table 2). Wynen et al. [32] found a similar result, placing *A. gazella *as sister to the *A*. *australis *+ *A. forsteri *+ *A. galapagoensis *clade, but placed *A. tropicalis *as sister to *A. pusillus *on a more basal branch separate from other arctocephaline species. Our results also support a polyphyletic *Arctocephalus*, but with *A. pusillus *as the separate lineage. The separation of *A. pusillus *from other *Arctocephalus *species (and possible pairing with *Otaria *as found in both the supermatrix analyses and the combined mtDNA supertree) is perhaps not unexpected in hindsight, given that this species has long been considered as having an 'enigmatic taxonomic position' due to its similarity to sea lions in size, skull morphology, and behaviour [64-66].

Several authors [31,32] have recently questioned the status of *A. philippii *and *A. townsendi *as separate species (also see [67,68]). Brunner [31] went so far as to suggest that both taxa be removed from *Arctocephalus *to form subspecies in the previously described genus *Arctophoca *(*Arctophoca philippii philippii *and *A. p. townsendi *[69]). Our results are equivocal on this latter issue, given that these two taxa do form part of the main clade of *Arctocephalus*, but as sister to the remaining species. The two taxa, however, are indicated to have diverged from one another earlier (0.3 mya; relDate date) than other another pair of undisputed *Arctocephalus *species (namely *A*. *gazella *and *A*. *tropicalis *at 0.1 mya), a potential argument in favour of them retaining separate species status (regardless of the generic appellation).

The close genetic relationship we found between *A. australis*, *A. forsteri *and *A. galapagoensis *(also [32]) is also congruent with the morphometric results of Brunner [31], who suggested that *A. galapagoensis *be considered a subspecies of *A. australis *(as per [66,67]). Again, the relatively long divergence time between these two taxa (0.7 mya; relDate date) could argue against this arrangement.

Ultimately, relationships within *Arctocephalus *remain poorly resolved with little agreement between different studies or, as shown in this study, even different analyses of the same base data set. This situation will likely remain at least until additional genes for these taxa are sequenced. We would note that the relationships and divergence times within *Arctocephalus *presented here are based on the only genetic marker available at the time data were extracted from GenBank (*MT-CYB *sequences [32]). Additional genetic sequences for these species are desperately required (but see [14,15]).

#### Divergence dates

The only recent studies to estimate divergence dates for otariids are those of Bininda-Emonds et al. [23] and Arnason et al. [14]. Here, we compare our estimates to those two studies and the available fossil record, which is unfortunately limited. Our relDate estimate of 8.2 ± 2.09 mya for the root of the otariid crown-group is younger than other recent estimates [14,23]. The multidivtime dates (ca. 11–12 mya) agree well with Bininda-Emonds et al. [23], but are still younger than that estimated by Arnason et al. [14]. Thereafter, a series of rapid divergences are inferred to have occurred. The origin of *Neophoca *was estimated at 6.1 mya based on *MT-CYB *only (ca. 10 mya using multidivtime), followed by the initial radiation of the remaining species at 5.2 ± 1.09 mya (ca. 9 mya using multidivtime), and the origins of *Otaria *at 4.5 ± 0.21 mya and *Arctocephalus pusillus *at 4.3 mya (the latter, again, based only on *MT-CYB*; both divergences ca. 7 mya in the multidivtime analyses). The oldest known record for the southern hemisphere otariids is established by *Hydrarctos lomasiensis *from the late Pliocene or early Pleistocene (< 3.4 mya, [70,71]). Fossils from California and Japan suggest that sea lions did not diversify until ca. 3 mya [55,56,72]; however, only the late Pleistocene occurrences (< 0.8 mya) of *Otaria bryonia *[73] and *Neophoca palatine *[74] are considered reliable at present [2]. Our date for the origin of the lineage leading to *Otaria *as a whole is naturally much older than this and almost three times older than that in Bininda-Emonds et al. [23] (which places *Otaria *in a very different position). Arnason et al. [14] estimated an older divergence time, but also based on a different phylogeny. We infer *Phocarctos *to have split from the remaining *Arctocephalus *species 3.4 ± 0.34 mya. Finally, the divergence between *Eumetopias *and *Zalophus *was dated as 4.5 ± 0.37 mya, which is considerably older that the earliest known fossils (Pleistocene, 1.64-0.79 mya [56]), but younger than the 8 mya estimate of Arnason et al. [14] (which is still older than the multidivtime estimate of ca. 6 mya).

Our results similarly indicate a rapid radiation within *Arctocephalus*, with many species originating within the past 1 million years (both dating methods, Tables 2, 3). Overall, the date estimates showed reasonable levels of variation (relDate median CV of 27.5), although some were highly variable. For example, the split between the clades *A. gazella *+ *A. tropicalis *and *A*. *australis *+ *A. forsteri *+ *A. galapagoensis *had a final date estimate of 3.1 mya but a large SE (3.43 my) and 95% confidence intervals on the input date (-2.76–10.68 mya), possibly reflecting weak signal in this area of the tree (see sensitivity analyses). Arctocephaline species are known in the fossil record only from poorly documented records of *A. pusillus *and *A. townsendi *from the Pleistocene (< 0.8 mya) [29]. The origin of *Arctocephalus *+ *Phocarctos hookeri *was estimated here using *MT-CYB *data at 4.3 mya, which is younger than other recent estimates based on different topologies [14,23]. Although our results lend support to previous suggestions [2,32] that both sea lions and *Arctocephalus *underwent recent periods of rapid radiation, all the evidence to date tend to be based on a small dataset for most species.

### Phocidae

#### Phylogeny

Compared to otariids, phocid relationships are generally much more agreed upon. The traditional and well-accepted phocid subfamilies Monachinae and Phocinae were both recovered as monophyletic in the supertree and supermatrix analyses (also see [4,11-15,23,26]). *Erignathus barbatus *was sister to the remaining northern phocids, followed by *Cystophora cristata*. The next branch of the tree separated *Pagophilus groenlandicus *and *Histriophoca fasciata *(= Histriophocina) as the sister group to the remaining taxa (but note the differences in the alternative supertree and the BI supermatrix). Most recent studies [11-15,23,26] have found support for this arrangement among the early branches (i.e., involving the lineages *Erignathus*, *Cystophora*, and Histriophocina). Of the six *Pusa*, *Phoca*, and *Halichoerus *species (= Phocina), in the preferred tree *Pusa hispida *was found to be sister to the remaining species in which *Phoca vitulina *+ *Phoca largha *formed the sister clade to (*Pusa sibirica *+ (*Halichoerus *+ *Pusa caspica*)) (again note the alternative arrangements in Figures 2 and 3, indicating poor signal in this part of the pinniped phylogeny). The sister-group relationship between *Phoca vitulina *and *P. largha *recovered here in all analyses is consistent among and well supported in numerous studies based on diverse data types [4,11-15,23,26], and reflects early suggestions that the latter species represents a subspecies of the former [68,75].

Arguably the biggest outstanding problem in phocid phylogeny concerns the placement of *Halichoerus *within Phocina, and there have been long-standing suggestions (e.g., [76]) for taxonomic revision of these taxa. Both Davis et al. [11] and Delisle and Strobeck [12] found the strongest support for *Halichoerus *as sister to *Pusa*, with both being sister to *Phoca*. However, both studies included only *Pusa hispida *as an exemplar for *Pusa*. Fulton and Strobeck [15] also recovered a similar result, but did not include *Pusa sibirica*. Four recent studies have included all three *Pusa *species [4,14,23,26]. Bininda-Emonds and Russell [4] recovered *Halichoerus *as sister to *Erignathus *+ Histriophocina + the remaining Phocina using morphological data. Bininda-Emonds et al. [23] resolved an unresolved *Pusa *as sister to the two *Phoca *species in their supertree, with *Halichoerus *being sister to this clade. The molecular results of Arnason et al. [14] and Palo and Väinölä [26] were similar to ours, indicating weak support for a *P. caspica *+ *H. grypus *clade, and for a basal position for *P. hispida *within Phocina. Although the precise interrelationships of the species differ slightly, our results support the suggestions of these other recent studies that both *Halichoerus *and *Pusa *be included within a redefined *Phoca*, possibly as subgenera. In fact, Arnason et al. [6] suggested recently that the scientific name for the grey seal be *Phoca grypa*. This solution also works in light of the continuing uncertainty concerning interrelationships within Phocina (compare Figures 1, 2, and 3 and these with the references above), especially the increasing number of suggestions that *Pusa *might be paraphyletic (except if it were to be retained as a subgenus).

It is also noteworthy that all the relevant divergences within Phocina apparently occurred in a very short time frame (also see [14,26]), which might make resolution within this group difficult to obtain even with additional markers. By contrast, there were no negative branch lengths in this part of the supertree (although nodes 23 and 24 in Figure 1 were held to be simultaneous initially), indicating relatively good agreement among the sequence data. Also, except for node 25, all the rQS values in this part of the (preferred) tree are > 0, again indicating more agreement than conflict among the set of gene trees (note the rQs values in Fig. 2, the only negative value in the alternative supertree concerns the sister-group relations of the two Histriophocina species).

Within Monachinae, all analyses recovered a monophyletic *Monachus *as sister to Miroungini + Lobodontini. Relationships within *Monachus *and *Mirounga *recovered here are consistent among and well supported in numerous studies [4,11-15,23,26] (but see [22] regarding *Monachus*). Relationships within Lobodontini have traditionally been contentious, although recent studies [11-15] all support the sister relationship between *Leptonychotes *and *Hydrurga *recovered here (contra [4,23]). However, the positions of *Ommatophoca *and *Lobodon *relative to each other and to the *Leptonychotes *+ *Hydrurga *clade remain problematic. Many recent studies [11,12,14,15] found the strongest support for an (*Ommatophoca*, (*Lobodon*, (*Leptonychotes *+ *Hydrurga*))) relationship. Our results differed and, similar to Fyler et al. [24], supported *Lobodon *as being sister to the remaining species. The supermatrix analyses indicated the identical sets of relationships for Monachinae.

#### Divergence dates

The fossil record suggests that the divergence of the two phocid subfamilies occurred sometime prior to the middle Miocene (> 14.6 mya) [77] and we used 16 mya as a minimum age constraint for the corresponding node (also see [23]). Similarly, Fyler et al. [24] used 15 and 17 mya as calibration points from which to estimate divergence dates in Monachinae. The corresponding molecular estimate of Arnason et al. [14] at 22 mya is older still and in better agreement with our multidivtime dates. The initial divergence in phocines (i.e., the lineage leading to *Erignathus*) was dated at 13.0 ± 0.90 mya, which is slightly younger than other estimates [14,23,24,26] (the multidivtime dates are again older, ca. 19 mya). Our relDate dates for the origins of *Cystophora *(8.0 ± 0.42 mya) and *Histriophoca *+ *Pagophilus *(6.4 ± 0.40 mya) are considerably younger than the corresponding estimates from Bininda-Emonds et al. [23] (which are in closer agreement with the multidivtime dates), but considerably older than the available fossil evidence. Deméré et al. [2] suggested that these basal phocines originated in the Arctic during the Pleistocene and represent the products of a glacioeustatic-forced allopatric speciation event. Arnason et al. [14] estimated a considerably older date (12 mya) for the divergence of *Cystophora*, again in agreement with both Bininda-Emonds et al. [23] and our multidivtime results, but a comparable 7 mya estimate for the origin of Histriophocina.

The genus *Phoca *arose 2.2 ± 0.62 mya (using relDate; multidivtime dates ca. 5–6 mya), with both extant species diverging from one another 1.1 ± 0.18 mya. These two nodes were well sampled, with 18 and 12 molecular estimates, respectively. The suggested recent separation and evolution of the two *Phoca *species (using both dating methods) is in general agreement with other studies [14,23,68,75,78]. *Pusa sibirica *arose 2.1 ± 0.21 mya, and *Halichoerus grypus *and *Pusa caspica *diverged immediately thereafter at 2.0 ± 0.14 mya; the divergence estimates for these last two nodes were each dated by only three genes apiece, and both are considerably older in the multidivtime analyses. Bininda-Emonds et al. [23], by contrast, estimated the origin of *Halichoerus *to be 7.1 mya, although this was based on a different topology, with *Halichoerus *in a more basal position. They also dated a *Pusa *polytomy to 2.8 mya, whereas we estimate here (using relDate) that the three genera *Phoca*, *Halichoerus*, and *Pusa *all arose over a short time span ranging from 2.2 to 1.1 mya (2–6 mya using multidivtime). Palo and Väinölä [26] similarly estimated that the radiation of the five main Phocini mtDNA lineages occurred ca. 2.5–3.1 mya, whereas Arnason et al. [14] estimated that the basal Phocina radiations occurred at 4.5 mya. Sasaki et al. [79] derived considerably younger estimates for divergences within *Pusa*, although their calibration was based on an incorrect estimate of the general mammalian substitution rate [26]. In addition, the sister-group relationships on which their dates are based conflict with our results and those of other recent studies [14,26]. Regardless of the precise relationships upon which the dates are based, the general consensus is that the diversification within Phocina was both rapid and relatively recent, which agrees with biogeographic evidence suggesting that the evolution of the *Halichoerus-Pusa-Phoca *complex likely occurred in the Greenland Sea/Barents Sea portion of the Arctic [2], and was possibly related to the closing of the Panama Canal 3.2-2.8 mya, which resulted in the freezing over of the Arctic Ocean [80-82].

Among the southern phocids, most nodes (with the obvious exception of the *Monachus schauinslandi *and *M. tropicalis *split) were well sampled, with 12–21 date estimates each. The lineage leading to *Monachus *split from the remaining species 11.3 ± 0.60 mya, which is slightly younger than other recent estimates [23,24] (and these other estimates are themselves slightly younger than the multidivtime dates). Our relDate estimate of the origin of the lineage leading to *M. monachus *(9.9 ± 0.28 mya) is considerably older than the 4.8 mya estimate of Bininda-Emonds et al. [23], but in good accord with those of Fyler et al. [24] and Arnason et al. [14]. The multidivtime dates for this node are again older, ca. 15–16 mya. The split between *M. schauinslandi *and *M. tropicalis *was interpolated to be 4.9 mya, compared to 2.8 mya estimate from Bininda-Emonds et al. [23] (also based on interpolation from a pure-birth model).

Our results indicate that the *Mirounga *lineage split from the lobodontine seals 10.0 ± 0.65 mya (ca. 15–16 mya using multidivtime), which accords well with recent genetic studies [14,23,24] and with fossil evidence indicating that the oldest fossils of southern lobodontines are from the late Miocene (6.7-5.2 mya) [71] and suggesting that the divergence occurred sometime before 11 mya [2,83]. Our relDate date for the split between the two *Mirounga *species (2.3 ± 0.85 mya) was slightly younger than that in other recent studies [14,23,24] (which were all in general agreement with the multidivtime results), but considerably older than the 0.8 mya estimate of Slade et al. [84].

Among the four lobodontine seals, *Lobodon *diverged first at 7.1 ± 0.34 mya, followed shortly thereafter by *Ommatophoca *at 6.8 ± 0.26 mya, and finally by *Hydrurga *+ *Leptonychotes *at 4.3 ± 0.55 mya. The time of origin of the lineage leading to *Lobodon *is younger than the date estimated by Fyler et al. [24], but older than that of Arnason et al. [14] (who also resolved a different topology). However, both it and time of origin of the lineage leading to *Ommatophoca *correspond well to the dates of Bininda-Emonds et al. [23]. The divergence dates determined using multidivtime were again considerably older (Table 3).

## Conclusion

Our results add to the growing list of studies that highlight the need for a re-evaluation of pinniped taxonomy, with revisions being required for both otariids (with respect to subfamilial classification and the genus *Arctocephalus*) and phocids (within Phocina, especially as regards *Halichoerus *and *Pusa*), ideally based on additional and especially nuclear genetic markers. The divergence-date estimates herein indicate rapid radiations in both families, particularly the southern hemisphere fur seals (*Arctocephalus*) and the northern phocids (Phocina), a fact which might account for the historical difficulties in assessing the phylogenetic relationships within each group. The historically unusual, but increasingly suggested nesting of *Halichoerus *within *Pusa *(see also [6,14,15,26]) highlights both the utility of large molecular datasets with numerous genes and the value of including all relevant species in phylogenetic analysis (see also [4]). We suggest increased genetic sampling throughout the group as the best approaches to further improving our understanding of pinniped phylogenetics. For example, at the time we gathered data, only *MT-CYB *had been sequenced for most otariid species and only a small number of genes were available for several *Pusa *species, although additional sequences have since been provided [14,15]. That being said, the problem areas within Phocina and *Arctocephalus *that were identified by both supertree and supermatrix analyses might prove resistant to resolution even with increased sampling should the apparent rapid branching in these parts of the tree be real.

Phylogenetic comparative methods have become the standard way for comparing aspects of the biology of a group of species while avoiding statistical problems associated with species not being independent due to their shared evolutionary history [85]. Phylogenetic analyses are improved with appropriate reconstruction of ancestral nodes using divergence-date information [86,87], and estimates of divergence dates provide conservation biology with a powerful tool in assessing vulnerability to conservation problems and comparative analysis of extinction risk [88,89]. Our results will allow the use of phylogenetic comparative methods with a robust estimate of pinniped phylogeny and divergence times that includes all species.

## Methods

### DNA sequence data

The use of large, multigene data sets provides the numerous informative changes required for correct inferences, and may also help to raise weak phylogenetic signals above the noise level [90]. In addition, the best topologies are often resolved when estimates are based on a combination of mitochondrial and nuclear DNA. With these points in mind, we mined GenBank for all available pinniped DNA sequence data to infer a phylogeny based on the largest data set possible. All sequence data were downloaded on January 30, 2006 and mined using the Perl script GenBankStrip v2.0 [91] to retain only those genes that had been sequenced for at least three pinniped species and were longer than 200 bp (except for tRNA genes, which had to be longer than 50 bp). For the 52 genes meeting these criteria (see Table 4), matching sequences for exemplars from Canidae (either *Canis lupus *or, on one occasion, *C. latrans*) and/or Ursidae (usually *Ursus arctos*, but also *U. americanus *or *U. maritimus *as needed) were downloaded for outgroup analysis.

**Table 4:** Genetic sequences used in this study with their inferred models of evolution.

			Phylogeny estimation		Fitting to supertree topology						
								
Gene	Number of taxa	Number of bps	Model selected	Nonclock ln L	Model selected	Nonclock ln L	Clock ln L	Chi-squared	df	LRT P-value	Clock?
*ALDOA*	7	120	K80 *	228.5901	K80	228.5901	228.98093	0.78166	5	0.9782	yes
*ALDOC*	7	129	K3P *	213.1006	K81+I	216.4168	218.7521	4.6706	5	0.4574	yes
*APOB *(editing region)	5	175	TVM *	317.7049	TVM+G	317.3427	319.90041	5.1154	3	0.1635	yes
*APOB *(exon 26)	6	963	HKY	1701.6729	HKY	1701.673	1705.76283	8.1797	4	0.08521	yes
*APOB *(exon 29)	3	621	n/a	n/a	TVM+G	1380.1937	1381.96031	3.5332	1	0.06015	yes
*CYP1A1*	5	1560	HKY	2641.2015	HKY+I	2913.9797	3546.2776	1264.6	3	0	
*CYP1A2*	5	1539	TVM+G	2859.5616	TVM+I	2861.4082	2863.05456	3.2927	3	0.3487	yes
*H2AFZ*	7	52	TrNef *	95.5468	TrNef	97.6302	99.00264	2.7449	5	0.7392	yes
*HLA-DOA*	5	399	HKY *	598.9182	HKY	598.9182	599.15301	0.46962	3	0.9255	yes
*LEP*	5	504	GTR *	1110.6539	GTR	1110.6539	1126.3367	31.366	3	7.12E-07	
*LYZ*	3	447	n/a	n/a	K80	677.5543	677.71442	0.32024	1	0.5715	yes
*MHC-DQA1*	7	162	TVMef *	393.3731	TVMef	393.7065	395.79033	4.1677	5	0.5255	yes
*MHC-DQA2*	7	230	HKY+G *	455.7005	HKY+I	458.4345	460.01745	3.1659	5	0.6744	yes
*MHC-DQB1*	5	141	K3Puf+G *	307.2022	TVM+I	306.0257	306.25311	0.45482	3	0.9287	yes
*MT-ATP6*	22	681	GTR+I+G *	4821.8578	TVM+I+G	4823.9243	4837.61057	27.373	20	0.1251	yes
*MT-ATP8*	22	204	HKY+I+G *	1598.4148	TVM+I+G	1597.1221	1609.01567	23.787	20	0.2518	yes
*MT-CO1*	25	1545	GTR+I+G *	10168.6228	TVM+I+G	10171.9424	10194.06882	44.253	23	0.004901	yes
*MT-CO2*	25	687	HKY+I+G *	4427.0054	HKY+I+G	4428.0015	4446.36885	36.735	23	0.03465	yes
*MT-CO3*	22	784	HKY+I+G *	4896.904	TVM+I+G	4893.5391	4909.02367	30.969	20	0.0556	yes
*MT-CYB*	35	1140	TrN+I+G *	8835.9098	GTR+I+G	8836.9434	8872.23839	70.59	33	0.0001522	
*MT-ND1*	22	957	HKY+I+G *	5866.8206	TVM+I+G	5863.5283	5882.62305	38.19	20	0.008394	yes
*MT-ND2*	24	1044	TrN+I+G *	7689.7091	TIM+I+G	7698.3613	7720.88041	45.038	22	0.002625	yes
*MT-ND3*	22	350	HKY+G *	2365.8095	TVM+I+G	2363.5559	2374.43717	21.763	20	0.3535	yes
*MT-ND4*	21	1378	GTR+I+G *	9587.3198	TVM+I+G	9586.7549	9609.42621	45.343	19	0.0006135	
*MT-ND4L*	22	300	HKY+I+G *	1900.4839	HKY+I+G	1897.8574	1909.16103	22.607	20	0.3085	yes
*MT-ND5*	22	1836	GTR+I+G *	13444.3742	TVM+I+G	13450.124	13482.66844	65.089	20	1.13E-06	
*MT-ND6*	13	528	HKY+I+G *	2457.8525	HKY+I+G	2457.8848	2466.38674	17.004	11	0.1078	yes
*MT-RNR1*	18	984	GTR+G *	4033.2182	GTR+I+G	4033.6277	4047.13902	27.023	16	0.04123	yes
*MT-RNR2*	14	1608	GTR+I+G	5722.1966	GTR+I+G	5710.8447	5730.04437	38.399	12	0.0001321	
*MT-TA*	7	69	HKY+G *	193.586	HKY+G	192.1818	197.11463	9.8657	5	0.07913	yes
*MT-TC*	7	69	K80+G *	190.9928	K80+G	190.9817	192.76403	3.5647	5	0.6136	yes
*MT-TD*	7	68	HKY+G *	177.8663	HKY+G	177.7805	178.50213	1.4433	5	0.9195	yes
*MT-TE*	9	72	HKY+G *	205.1879	HKY+I	204.3486	208.84935	9.0015	7	0.2525	yes
*MT-TF*	7	72	TrNef *	201.267	TrN+G	197.1401	202.84986	11.42	5	0.04366	yes
*MT-TG*	7	72	TrNef+G *	269.0899	HKY+G	267.3327	271.41557	8.1657	5	0.1473	yes
*MT-TH*	7	69	HKY+G *	201.8982	HKY+G	203.5126	205.11176	3.1983	5	0.6694	yes
*MT-TI*	7	71	HKY+G *	141.7316	HKY+I	142.4105	145.12331	5.4256	5	0.3662	yes
*MT-TK*	7	70	HKY+G *	219.0096	HKY+G	221.4528	228.78366	14.662	5	0.01191	yes
*MT-TM*	7	71	K80+G *	140.5878	TrNef+I+G	135.8801	145.67015	19.58	5	0.001498	yes
*MT-TN*	7	73	K80+G *	204.0605	HKY+I	201.8885	205.49416	7.2113	5	0.2054	yes
*MT-TP*	8	68	HKY+G *	200.9591	HKY+G	202.8423	204.98693	4.2893	6	0.6376	yes
*MT-TQ*	7	76	HKY+G *	202.9076	TrN+G	203.4861	222.92371	38.875	5	2.52E-07	
*MT-TR*	8	71	HKY+G *	197.8915	K81uf+G	196.7379	201.8366	10.197	6	0.1166	yes
*MT-TT*	9	74	K80+G *	226.9842	HKY+I	224.7003	230.34978	11.299	7	0.1261	yes
*MT-TV*	8	76	K80+G *	231.1146	HKY+G	230.1562	233.04494	5.7775	6	0.4486	yes
*MT-TW*	7	68	K80+I+G *	215.6285	HKY+I+G	211.9622	218.25694	12.589	5	0.02755	yes
*MT-TY*	7	70	K80+G *	225.9578	HKY+I	222.7287	230.7563	16.055	5	0.006689	yes
*MX1*	4	1980	TrN+G	3887.1331	TrN+G	3887.1331	3892.79094	11.316	2	0.003489	yes
*RAG1*	5	741	HKY+G	1713.0793	TVM+I	1709.8627	1712.53176	5.3381	3	0.1486	yes
*RHO*	5	1077	HKY+I+G	2055.8504	HKY+I	2056.0244	2056.56021	1.0716	3	0.7839	yes
*SERPINA7*	7	442	TrNef *	869.6311	TrNef	869.631	870.82096	2.3799	5	0.7945	yes
*SRY*	21	231	HKY *	488.8158	HKY	488.3143	494.82962	13.031	19	0.837	yes
											
TOTAL		26818									

Models followed by an asterisk were determined using AICc; all others were determined using AIC. *LYZ *and *APOB *(exon 29) were not used to construct the supertree. Gene symbols follow Wain et al. [126].

Sequences in each data set were aligned using ClustalW [92] or with transAlign [93] in combination with ClustalW for the protein-coding sequences, and improved manually where needed. Thereafter, each aligned data set was passed through the Perl script seqCleaner v1.0.2 [91] to standardize the species names, to eliminate inferior sequences (i.e., those with > 5% Ns), and to ensure that all sequences overlapped pairwise by at least 100 bps (or 25 bps for the tRNA genes). Note that although species names were standardized according to Wilson and Reeder [94] for the analyses, those used in the text for Phocini follow the currently accepted International Commission of Zoological Nomenclature (ICZN) taxonomy, which recognizes the five genera *Halichoerus*, *Histriophoca*, *Pagophilus*, *Phoca*, and *Pusa*.

The final data set of 52 genes (Table 4) comprises 26818 bps in total, or an average of 515.7 bp per gene (range = 68–1980 bps). On average, each gene was sampled for 11.2 species (range = 3–35); however, only an average of 5.5 species per nuclear gene were available for study. Two genes, *LYZ *and exon 29 of *APOB*, contained fewer than three pinniped species and, as such, were uninformative for resolving pinniped interrelationships. However, they were still retained to determine times of divergence. Accession numbers for all sequences used in the final data set are provided as supplementary material (Additional file 1).

The final data set is dominated by mitochondrial genes, which forms a single locus due to its common inheritance and general lack of recombination. As such, it must be kept in mind that all the resulting topologies (be they derived in a supertree or supermatrix framework) and divergence times could be biased by any peculiarities related to mitochondrial sequence data (e.g., introgression or linkage) or simply the disproportionately large amount of mitochondrial data. However, the data set represents the "current systematic database" for pinnipeds and so the best possible current data source for which to infer their phylogenetic relationships. However, to assess the impact of this potential source of bias, we performed a second supertree analysis where all mtDNA genes were combined to form a single source tree (yielding 12 source trees in total). Nevertheless, the collection of additional nuclear markers is desperately needed for this group.

The final data set used for the phylogenetic analyses, together with the supertree and supermatrix trees is freely available from TreeBASE [95] (study accession    number S1911, matrix accession numbers M3516-M3518).

### Phylogeny reconstruction and supertree analysis

Our general approach to infer the phylogeny of the pinnipeds involved a divide-and-conquer strategy in which individual genes trees were determined using the best possible methodology for each and then combined as a supertree. Compared to a simultaneous analysis of the multigene "supermatrix", this procedure has been argued to potentially account better for the differential models of evolution that might be present [96] and, for extremely large matrices, looks to be a faster analytical method without any appreciable loss of accuracy [97]. Although the use of mixed models is possible in both maximum likelihood (ML, [98]) and Bayesian frameworks, the accuracy of the resulting tree, at least in a Bayesian framework, has recently been called into question [99], especially when reasonable levels of conflict exist between the different data partitions [100]. Furthermore, Jeffroy et al. [101] have also recently argued that trees derived from multigene, phylogenomic data sets should be treated more cautiously than those from single-gene analyses given that the systematic biases inherent to phylogeny reconstruction become more apparent with larger data sets. Nevertheless, in light of the fierce criticism that the supertree approach has attracted (e.g., [102,103], but see [104,105]), we also conduct ML and Bayesian inference (BI) analyses of the concatenated supermatrix to help identify especially problematic regions of the pinniped tree as part of a global congruence framework [50] and to add to the growing body of studies comparing phylogenetic inference under these two frameworks (e.g., [15,106]).

For the supertree analyses, we used PHYML [106] to determine the ML tree for each of the 50 phylogenetically informative genes after determining their optimal model of evolution according to either AIC or AICc (as appropriate, the latter being a version of the AIC corrected for small sample sizes) using MrAIC [107] and PHYML [106] (Table 4). The 50 gene trees were then used to build a weighted supertree of the group using matrix representation with parsimony (MRP, [48,49]). In so doing, we have assumed that each gene tree forms an independent unit in our preferred supertree, something that is admittedly debatable for the mitochondrial genes and especially the very small tRNA genes. However, in the absence of any robust linkage information, this assumption seemed more justifiable and objective than the defining of gene partitions based on assumed linkage or for purely practical considerations (e.g., concatenating all the tRNA genes because of their small size). Nonetheless, the sensitivity of these assumptions was assessed using the second supertree in which all mtDNA genes formed a single source tree.

All gene trees were encoded for the MRP analysis using semi-rooted coding [108], whereby only those trees with either a canid and/or ursid outgroup taxon and where the pinnipeds were reconstructed as being monophyletic were held to be rooted. Furthermore, the individual MRP characters, which correspond to a particular node on a gene tree, were weighted according to the bootstrap frequency [109] of that node, as determined using PHYML and based on 1000 replicates. This procedure has been demonstrated to increase the accuracy of MRP supertree construction in simulation [110]. The weighted parsimony analysis of the resulting MRP matrix was accomplished using a branch-and-bound search in PAUP* v4.0b10 [111], with Canidae and Ursidae being specified as a paraphyletic outgroup. *Monachus tropicalis*, for which no molecular data exist, was added to the supertree manually as the sister species of *M. schauinslandii *(following [4,23]).

Support for both supertrees and the relationships in them were quantified with the supertree-specific rQS index [51,52], which compares the topology of the supertree to that of each of the source trees contributing to it. As such, it is preferable to such conventional, character-based support measures such as Bremer support [112] and the bootstrap, which are invalid in this context given that MRP characters for a given source tree are non-independent. Values for rQS range from + 1 to -1, with the two values indicating that a given node is directly supported or directly contradicted by all source trees, respectively. The rQS value for the entire tree is simply the average of all the nodal rQS values. Previous applications of the rQS index show that it often tends to negative values [51,52,113], indicating that more conflict than agreement generally exists among a set of source trees for a given node. As such, positive values of rQS can be taken to indicate good support in the sense that more source trees support the relationship than contradict it.

The individual gene data sets were also concatenated to form a single supermatrix that was analyzed using both partitioned ML and BI methods. ML analyses used RAxML VI-HPC v2.2.3 [114]. A GTR + G model was assumed for the data using the CAT approximation of the gamma distribution, with the model parameters being allowed to vary independently for each gene. CAT is both a fast approximation of the gamma model (due to its lower computational and memory costs) and one that appears to yield better log likelihood scores even when calculated under a real gamma model [115], and therefore is ideally suited to large, computationally intensive data matrices such as ours. The ML tree was taken to be the optimal tree over 100 replicates, for which nodal support was estimated using the bootstrap with 1000 replicates and search parameters matching those for the optimality search.

BI used MrBayes v3.1.2 [116], with the individual models specified for each individual gene matching the optimal model determined in the gene-tree analyses as closely as possible. Otherwise, flat priors were used in all cases. Searches employed a MCMC algorithm of two separate runs, each with four chains that were run for 10000000 generations and with the first 5000000 generations being discarded as burn-in. Trees were sampled every 5000 generations to derive the final BI tree and estimates of the posterior probabilities.

### Divergence date estimations

Following Bininda-Emonds et al. [117], divergence times on the supertree only were determined using a combination of fossil calibration points and molecular dates under the assumption of a local molecular clock (see [118]). As a first step, the optimal model of evolution for all 52 genes was (re)determined using an AIC in ModelTEST v3.6 [119] in combination with PAUP*, with the appropriately pruned supertree topology being used as the reference tree in place of the default NJ tree. This combination was used here in place of the previous MrAIC/PHYML combination largely because it can be used to test for the applicability of a molecular clock (through PAUP*) using a likelihood-ratio test. The small taxonomic distribution meant that all but six genes (*CYP1A1, MT-ND4, MT-ND5, MT-RNR2, OB*, and *MT-TQ*) evolved according to a molecular clock.

Thereafter, we used PAUP* to fit the sequence data for each gene to the (pruned) supertree topology under the optimal model in a ML framework. In line with Purvis' [118] local-clock model, the relative branch lengths for each gene tree relative to the topology of the supertree were determined using the Perl script relDate v2.2.1 [91]. Only the gene trees for the clock-like genes were considered to be rooted and relative branch lengths were calculated with respect to ancestral nodes only (and not also with respect to daughter nodes).

Divergence times were then determined by calibrating the relative branch lengths for each gene tree using a set of fossil dates (Table 5). For a given node, the initial divergence date was taken to be the maximum of 1) the median of all fossil plus molecular estimates and 2) the fossil estimate. In this way, the fossil estimate acts as a minimum age constraint that can overrule the molecular estimates. Upper and lower bounds on any given date estimates took the form of the 95% confidence interval derived from all individual gene and/or fossil estimates for that node. Although error in the branch-length estimation for the individual gene trees can also contribute to uncertainty in the final date estimates [120], it is likely to be less important than the variation present between the different genes themselves. However, together with uncertainties in the fossil dates, it cannot be excluded that our confidence intervals are underestimates of the true values.

**Table 5:** Fossil calibrations used to anchor molecular date estimates.

Divergence	Date	Source(s)	Node
Canids from arctoid carnivores	43.5	[127]	1
Pinniped and ursid split	19.5	[17]	2
Split between Phocidae and Otariidae + Odobenidae	23	[2]	3
Odobenoids first evolved	18	[2]	4
Monachinae-Phocinae split	16	[1, 55, 128]	18
Split between *Monachus *and other southern phocids	8	[71, 128]	28
Split between *Mirounga *+ Lobodontini	7	[71, 128]	29
Origin of *Callorhinus*	6	[56]	5

All dates (in millions of years ago) also represent minimum age constraints.

Finally, the Perl script chronoGrapher v1.3.3 [91] was used to correct for any negative branch lengths and simultaneously to derive a divergence-time estimate for the single node lacking an initial estimate (that linking *Monachus schauinslandi *and *M. tropicalis*). The date for this latter node was interpolated from the dates of up to five of its ancestral nodes based on the relative number of species descended from each node, assuming a constant birth model (see [117]).

More details regarding this dating procedure, including its strengths and weaknesses with respect to other relaxed molecular clock methods (recently reviewed in [121]) can be found in Bininda-Emonds et al. [117].

The Bayesian relaxed molecular clock method implemented by multidivtime [122,123] was also used to calculate divergence dates from the supermatrix data fitted to the preferred supertree topology. General methodology followed Rutschman [124], with maximum likelihood parameters estimated using PAML version 3.15 [125]. Incomplete overlap of sequences between taxa (in particular the outgroup sequence(s) not being represented in every partition) meant that model partitioning by gene was impossible; instead, a single F84 + gamma model was applied to the entire supermatrix. The root prior rttm (the mean of the prior distribution for the time from the ingroup root to the tips; in other words, the age of the ursid-pinniped split) was specified as 19.5 mya, with the remaining constraints the same as in the supertree dating analysis (Table 5). Other multidivtime parameters were calculated following the recommendations of Rutschmann [124]: rtrate (mean of prior distribution for the rate at the root node) = X/rttm, where X is the median amount of evolution from the root to tips; rtratesd (standard deviation of rtrtate) = 0.5 × rtrate; brownmean (mean of the prior distribution for the autocorrelation parameter, *v*) = 1/rttm; brownsd (standard deviation of brownmean) = brownmean. Three independent multidivtime analyses were run for 1 × 10^6 ^cycles, with samples taken every 100 cycles after a burn-in period of 1 × 10^5 ^cycles. The dates presented here are mean values for the three runs. The multidivtime analyses were then repeated using only the mitochondrial genes to investigate whether the inclusion of nuclear genes greatly altered the estimated divergence dates.

## Authors' contributions

JWH collected data, conceived of and coordinated the study, assisted with analyses and drafted the manuscript. OB-E collected data; conducted the supertree, supermatrix, and dating analyses. RB conducted the multidivtime analyses and helped draft the manuscript. SHF and OB-E participated in the design of the study and helped draft the manuscript, and all authors read and approved the final manuscript.

## Supplementary Material

Additional file 1 GenBank accession numbers. GenBank accession numbers for all pinniped, canid and ursid sequences used in this study. Click here for file
