# Phylogeny and divergence of the pinnipeds (Carnivora: Mammalia) assessed using a multigene dataset

**DOI:** 10.1186/1471-2148-8-216

**Published:** 2008-07-24

**Authors:** Jeff W Higdon, Olaf RP Bininda-Emonds, Robin MD Beck, Steven H Ferguson

**Affiliations:** 1 Department of Geography, University of Manitoba, 501 University Crescent, Winnipeg, Manitoba, R3T 2N6, Canada; 2 AG Systematik und Evolutionsbiologie, IBU – Fakultät V, Carl von Ossietzky Universität Oldenburg, 26111 Oldenburg, Germany; 3 School of Biological, Earth and Environmental Sciences, University of New South Wales, Sydney NSW 2052, Australia; 4 Fisheries and Oceans Canada, 501 University Crescent, Winnipeg, Manitoba, R3T 2N6, Canada

## 

After publication of our article [1], we discovered that one of the programs used to date the supertree, relDate v2.2 [2], contained a bug that can lead to errors in the date estimates it provides. The precise effect of the bug is dependent on tree shape, but it generally results in overestimates of divergence-time estimates, particularly for nodes that are closer topologically to the tips of the tree. It can potentially, however, affect every date estimate on a tree.

Fortunately, the impact of the bug on date estimates in the pinniped supertree was minimal. Only 14 of the 35 nodes were affected, with the mean and median absolute errors for the dates of these 14 nodes being 0.19 and 0.10 million years (= 8.9% and 6.1% relative to the corrected date). All but one error (that for node 14) were overestimates. The largest difference was 0.5 million years (for node 6), and the two sets of dates are perfectly correlated with one another (correlation coefficient = 1.000, *Z *= 28.508, *P *< 0.0001).

Corrected dates and 95% confidence-interval dates for all nodes in the pinniped supertree can be found in Table 1 and should be used in place of those reported in the original publication [1]. We apologize for any inconvenience this error might cause.

**Table 1:** Published and corrected divergence dates for the world's pinnipeds as determined by the relDate method.

			Confidence-interval dates	
			
Node	Published date	Corrected date (SE)	Lower	Upper
1	43.4	43.4	43.4	43.4
2	35.7	35.7	30.6	40.9
3	23.0	23.0	20.3	25.7
4	18.0	18.0	15.3	20.7
5	8.2	8.2	6.0	12.3
**6**	6.1	5.6	4.8	6.5
7	5.2	5.2	4.0	6.3
**8**	4.5	4.4	3.9	5.4
**9**	4.3	4.2	3.7	5.2
**10**	3.4	3.3	2.7	5.1
**11**	3.2	3.1	2.4	5.0
**12**	3.1	3.0	0.6	4.9
13	1.1	1.1	0.5	1.6
**14**	0.7	0.8	0.3	0.8
15	0.1	0.1	0.2	0.1
16	0.3	0.3	0.3	0.3
17	4.5	4.5	3.5	5.3
18	16.0	16.0	14.2	17.8
19	13.0	13.0	11.2	14.7
**20**	8.0	7.9	7.1	8.7
21	6.4	6.4	5.6	7.1
**22**	2.4	2.1	1.6	2.9
**23**	2.2	1.9	1.4	2.7
**24**	2.1	1.8	1.2	2.3
**25**	2.0	1.7	1.1	2.0
**26**	1.1	0.9	0.6	1.3
27	4.3	4.3	3.3	5.3
28	11.3	11.3	10.1	12.5
**29**	10.0	9.9	8.7	11.2
30	7.1	7.1	6.4	7.7
31	6.8	6.8	6.3	7.3
32	4.3	4.3	3.2	5.4
33	2.3	2.3	0.6	4.0
34	9.9	9.9	9.4	10.5
35	4.9	4.9	4.4	5.3

Node numbers correspond to Figure 1 in Higdon *et al*. [1]; those where the dates have changed are listed in bold face. All dates (including 95% confidence-interval dates) are in millions of years ago.
